# Estimation of Polycyclic Aromatic Hydrocarbons Pollution in Mediterranean Sea from Volturno River, Southern Italy: Distribution, Risk Assessment and Loads

**DOI:** 10.3390/ijerph18041383

**Published:** 2021-02-03

**Authors:** Paolo Montuori, Elvira De Rosa, Fabiana Di Duca, Donatella Paola Provvisiero, Pasquale Sarnacchiaro, Antonio Nardone, Maria Triassi

**Affiliations:** 1 Department of Public Health, “Federico II” University, Via Sergio Pansini 5, 80131 Naples, Italy; elvira.derosa@unina.it (E.D.R.); fabianadiduca91@gmail.com (F.D.D.); donatellapaola.provvisiero@gmail.com (D.P.P.); antonio.nardone@unina.it (A.N.); triassi@unina.it (M.T.); 2 Department of Law and Economics, University of Roma “Unitelma Sapienza”, Viale Regina Elena 295, 00161 Rome, Italy; pasquale.sarnacchiaro@unitelma.it

**Keywords:** polycyclic aromatic hydrocarbons, Volturno River, composite indicator, contaminant loads, risk assessment

## Abstract

This study reports the data on the contamination caused by polycyclic aromatic hydrocarbons (PAHs) drained from the Volturno River. The seasonal and spatial distribution of PAHs in water and sediment samples was assessed. The 16 PAHs were determined in the water dissolved phase (DP), suspended particulate matter (SPM), and sediments. A multidimensional statistical approach was used to identify three pollution composite indicators. Contaminant discharges of PAHs into the sea were calculated in about 3158.2 kg/year. Total concentrations of PAHs varied in ranges 434.8 to 872.1 ng g^−1^ and 256.7 to 1686.3 ng L^−1^ in sediment samples and in water (DP + SPM), respectively. The statistical results indicated that the PAHs mainly had a pyrolytic source. Considering the sediment quality guidelines (SQGs), the water environmental quality standards (USEPA EQS), and risk quotient (RQ), the Volturno River would be considered as an area in which the environmental integrity is possibly at risk.

## 1. Introduction

PAHs are environmental pollutants commonly distributed in the ecosystem [1,2]. Their diffusion in the environment caused much concern due to the toxic and carcinogenic properties of these compounds for human health [3,4,5]. The PAHs release into the aquatic system is caused by both natural and human sources. Natural sources of PAHs include volcanic emissions, forest fires, natural oil, and certain biological (biogenic) processes. Anthropogenic sources of PAHs in aquatic environments are divided into pyrogenic and petrogenic groups. Pyrogenic sources include incomplete combustion, domestic and industrial wastewater discharges, oil spills and waste incinerators, etc. Petrogenic sources include crude oil and petrochemicals products such as kerosene, petrol, diesel, lubricating oil, and asphalt [6,7,8]. All of the aforementioned sources (i.e., pyrolytic, petrogenic and diagenetic) bring about distinctive PAHs patterns.

Combustion products are composed of two or more fused aromatic rings. In most cases, high molecular weight PAHs (HMW-PAHs) are dangerous and havethe tendency to bio-accumulate in the human body and different aquatic organisms, whereas low molecular weight compounds (LMW-PAHs)—such as those with bi- and tricyclic aromatic rings—are normally produced during low temperature diagenesis, which is in the process of oil production (i.e., petrogenic sources) [9,10,11]. Using specific chemicals analysis, identifying the generating processes of the sample is achievable [12,13].

**Figure 1:**
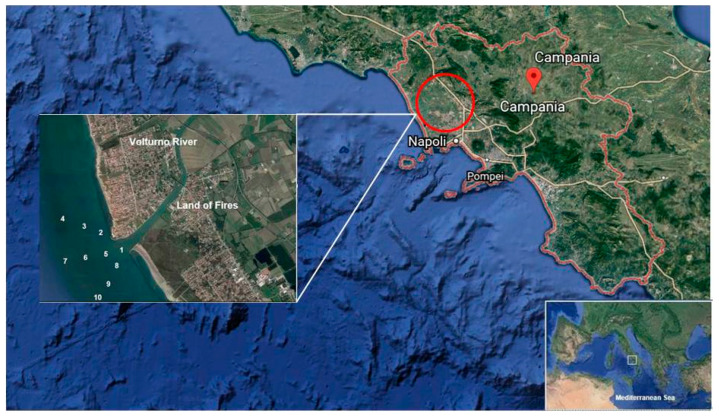
Map of the study area and sampling sites in the Volturno River and Estuary, Southern Italy. Source: Google Earth.

Since 1980, waste disposal in Campania region is a relevant problem. This waste problem in the Campania region caused a considerable discharge of illegal urban, toxic, and industrial waste [14,15]. In recent years, the Campania region was involved in some environmental emergencies: Illegal dumping and burning of toxic waste. Due to these events, this area has been renamed as “Land of Fire” (Figure 1). Frequently, extensive quantities of accumulated waste are illegally burned in the countryside and on the road, and the smoke released contains many PAHs [14,16].

Illegal waste disposal caused a significant environmental impact on water, air, and soil quality. In fact, it led to the deterioration of land and especially ground and surface water. The “Land of Fire”, also called “Triangle of Death” by Senior and Mazza in the journal The Lancet Oncology, is crossed by the Volturno River, which is the main basin of Southern Italy for length and water flow. Therefore, PAHs present in the toxic fumes from waste illegal fires, can deposit on the surface waters of the Volturno River which, consequently, can cause pollution of the Mediterranean Sea where Volturno River flows [17,18,19].

Estuarine and coastal marine environments receive a substantial input of PAHs due to having served as major depositories for the disposal of industrial and domestic effluents, sewage sludge, and dredged material with considerable loads contaminants. Water could represent a direct measure of the degree of aquatic environment. Sediments are natural sinks and environmental reservoirs for PAHs in the aquatic system and they provide an irreplaceable aid in reconstructing the input and contamination of PAHs.Due to their high persistence in the environment, PAHs accumulate in the sediment for a long time and may be a source of contamination for aquatic biota. This makes the PAHs presence in the water and sediment matrices a paramount issue with utmost attention for the aquatic environment, fishing, and seafood producing industries [20,21].

The purpose of this study is to report data concerning PAHs drainage contamination from the Volturno River and its impact on the Tyrrhenian Sea (Central Mediterranean Sea). Moreover, the ecosystem risk and the PAHs spatial and seasonal distribution in samples of water and sediment was evaluated in this river. One of the key aims of this study is to estimate the PAHs as indicators of contamination, by determining the main sources from which these pollutants originate. To the best of our knowledge, no previous studies estimate the PAHs input into the Central Mediterranean Sea from Volturno River.

## 2. Materials and Methods

### 2.1. Sampling 

Four sampling campaigns, each of which in 10 sites, were performed from November 2017 to July 2018, to evaluate the changing trends of PAHs in time. In Table 1, information about sampling sites are shown. Throughout sampling, a global positioning system (GPS) was used to locate the sampling positions and environmental data such as temperature, pH, and conducibility were measured in situ with a portable instrument (Mettler Toledo—SevenExcellence pH/Conductivity BenchtopMeter S470-Std-K) (Table S1).Site 1 was at the Volturno River mouth, while the other sites were at 500, 1000, and 1500 mt away from the Volturno Estuary, in order to assess the levels of the PAHs contamination and to estimate the impact of the river contamination on the Mediterranean Sea.

Water samples (2.5 L) were collected from the surface layer at the depth of 0–50 cm from the sampling locations using amber glass bottles directly and taken to the laboratory for analysis. Superficial sediments (at the depth of 0–5 cm) were collected in April with a handheld Van Veen Grab sampler, placed in aluminum containers and kept frozen (−20 °C) until further analysis. 

### 2.2. Sample Cleanup and Analysis

Water samples were filtered through GF/F glass fiber filters (0.7 μm pore size, Whatman, United Kingdom; baked at 450 °C overnight), then the filters (suspended particulate matter, SPM) were preserved at −20 °C until analysis.

In the dissolved phase (DP), PAHs were extracted with a solid phase extraction (SPE) by the Oasis HLB cartridge (6 mL, 500 mg; Waters, Milford, MA, USA), according to the method proposed by Zhou et al. [22]. Before extraction, benzo[a]pyrene-*_d12_* and indeno[1,2,3-cd]pyrene-*_d12_* were added as surrogate solutions (10 ng L^−1^). Methylene chloride (5 mL), methanol (5 mL), and ultra-pure water (5 mL) were used to condition and wash the cartridge, then the water sample (2 L) was eluted with a flow rate of 10 mL min^−1^. Therefore, the cartridge was eluted with methylene chloride (10 mL) and the extract was concentrated to 500 µL in hexane for the GC-MS analysis. Finally, chrysene-*_d12_* was added to the sample as an internal standard.

The sediment was air-dried, pulverized, sieved (250 µm particles were used as the sample) homogenized, and sub-sampled into 5-g portions. The PAHs concentrations in the sediment samples were calculated as dry weight (ng/g dw) [23,24,25,26].

The PAHs extraction from the filters (SPM) and the sediment samples was performed with a Soxhlet extractor using methylene chloride (150 mL) for 24 h, followed by 150 mL of acetone:hexane (1:1) solution for another 24 h. In the Soxhlet extraction, 5 g of the sediment sample was placed into an extraction thimble, which was then extracted using the solvent via the reflux cycle. Once the solvent was boiled, the vapour passed through a bypass arm into the condenser, where it condenses and drips back onto the solvent in the thimble. As the solvent reaches the top of the siphon arm, the solvent and extract were siphoned back onto the lower flask whereby the solvent reboils, and the cycle was repeated until all the samples were completely extracted into the lower flask. The extracts were purified via a column consisting of 1 g of sodium sulfate and 2.5 g (10% deactivated) of silica geland eluted with 70 mL of hexane:methylene chloride (7:3) solution. The extracts were evaporated to dryness, solvent-exchanged to hexane to 500 μL for the GC-MS analysis, and chrysene-*_d12_* was added as an internal standard. The total organic carbon (TOC) was determined by the TOC analyzer (TOC-VCPH, Shimadzu Corp., Kyoto, Japan).

### 2.3. Instrumental Analysis

A TRACE^TM^1310gas chromatographycoupled to the ISQ^TM^ 7000 single quadrupolemass spectrometer (GC-MS, Thermo Scientific, Waltham, MA, USA) under a selected ion monitoring (SIM) mode was used to perform the analysis. A TG-5MS capillary column (30 mm length × 0.25 mm inner diameter × 0.25 μm film thickness) and the helium as a carrier gas (constant flow rate of 1 mL/min) were used. TheMSD worked in the electron ionization (EI)mode, set at 70 eV. A splitless injection mode was adopted and the sample injection volume was 1 μL. The column temperature was programmed as follows: 60 °C for 2 min, increasing to 200 °C at 25 °C min^−1^, to 270 °C at 10 °C min^−1^ (kept for 6 min), and finally, to 310 °C at 25 °C min^−1^ (kept for 10 min). The injector and detector temperatures were 280 and 300 °C, respectively.

The PAHs quantification was made using a five-point calibration curve for the 16 PAHs (Dr. Ehrenstorfer GmbH, Augsburg, Germany) (r^2^ > 0.98) and chrysene-*_d12_* as an internal standard.

The following PAHs, according to the USEPA priority [27], were analyzed: Naphthalene (Nap), acenaphthylene (Acy), acenaphthene (Ace), fluorene (Flu), phenanthrene (Phe), anthracene (An), fluoranthene (Fl), pyrene (Pyr), benzo[a]anthracene (BaA), chrysene (Chr), benzo[b]fluoranthene (BbF), benzo[k]fluoranthene (BkF), benzo[a]pyrene (BaP), dibenzo[a,h]anthracene (DahA), benzo[ghi]perylene (BghiP), and indeno[1,2,3-cd]pyrene (InP). Perylene (Per) was also analyzed, although it is not present in the list of the priority pollutants proposed by USEPA. The total PAHs concentration was calculated as the sum of the concentrations of the above individual PAHs compounds (ΣPAHs).

### 2.4. Quality Assurance and Quality Control

To estimate the quality assurance and quality control of the applied method, blanks and spiked recoveries were determined during the sample collection, preservation, and analysis. Before the process, all the glassware was heated to 200 °C and then washed thoroughly with methanol, acetone, and dichloromethane to minimize the background pollution. The detection limit (LOD) was calculated as three times the noise in a blank sample chromatogram. A total of ten blanks were analyzed as the same as the samples; PAHs in the blanks showed a concentration below the LOD.

In the water, LODs ranged from 0.01 to 0.1 ng L^−1^ while, in SPM and sediment samples, from 0.03 to 0.2 ng L^−1^and from 0.01 to 0.15 ng g^−1^, respectively.

The quantification limits (LOQ) were in the range of 0.02–0.15 ng L^−1^ in the water samples, 0.06–0.3 ng L^−1^ in the SPM samples, and 0.03–0.2 ng g^−1^ in the sediment samples. Data below the LOD were presented as <LOD. The recovery of PAHs in standard checks and samples was between 70% and 130%, which met the quality control requirements.

### 2.5. Statistical Analysis and Calculation of PAHs Inputs

The statistical software SPSS, version 14.01 for Windows (SPSS Inc., Chicago, IL, USA) was used to analyze the data for the purpose of calculating the PAHs input. All data were presented as the mean ±standard deviation (SD) and only *p*-values≤ 0.05 were considered statistically significant.

First, the flow-averaged mean concentration (*C_aw_*) and the annual contaminant discharges (*F*_annual_), according to the UNEP guidelines [28]and widely accepted [29,30,31], were evaluated using the following equations:

(1) $$C_{aw}=\frac{\sum_{i=1}^{n}C_{i}Q_{i}}{\sum_{i=1}^{n}Q_{i}}$$

*F*_annual_ = *C_aw_Q*_T_ (2)

where *C_i_* is the instantaneous concentration, *Q_i_* and *Q*_T_ are the daily averaged water flow discharge and the total river discharge over the period considered (November 2017–July 2018), respectively [31,32,33]. The temporal pollutant discharge variation was evaluated considering *C_i_* and *Q_i_* for each campaign expressed as kg/year.

River flow data were collected from the register of the Autorità di Bacino Nazionale dei Fiumi Liri-Garigliano e Volturno to http://www.ildistrettoidrograficodellappenninomeridionale.it (Abruzzo, Basilicata, Calabria, Campania, Lazio, Molise, Puglia Government for the Environment).

The principal component analysis (PCA) allows reducing the size of a data set (sample) by finding a new smaller set of variables, that nonetheless retains most of the sample’s information [34,35]. There are three main methods used in order to determine the optimal number of components [36,37,38,39] in a principal component model (amount of explained variance, Cattell’s scree test, and Kaiser’s Eigenvalue greater than the 1.0 rule). In order to enhance the interpretation of the results of the PCA, it is possible to rotate the axes to reduce the dimensions or cover the maximum variation.

The rotation and change of the coordinates are performed by the Varimax rotation method. In order to analyze in depth the pollution of PAHs affecting the Volturno River and its environmental impact on the Tyrrhenian Sea, the principal component analysis has been conducted on a dataset obtained on DP and SPM. In each analysis, the 17 PAHs above mentioned were evaluated.

## 3. Results and Discussion

### 3.1. PAHs Concentrations in Water Samples

The amount of total PAHs in the DP, determined at 10 sampling locations during the four campaigns, ranged from 64.3 (site 8) to 1429.1 (site 1) ng L^−1^ with a mean value of 602.6 ± 319.3 ng L^−1^ (Table 1). These data ranged from 3.44 to 174.4 ng L^−1^ with a mean value of 58.4 ± 39.5 ng L^−1^ for 2-ring PAHs (Nap), from 19.9 to 805.1 ng L^−1^ for 3-ring PAHs (Acy, Ace, Flu, Phe, An), from 12.7 to 244.1 ng L^−1^ for 4-ring PAHs (Fl, Pyr, BaA, Chr), from 9.48 to 151.1 ng L^−1^ for 5-ring PAHs (BbF, BkF, BaP, DahA), and from 17.6 to 74.6 ng L^−1^ for 6-ring PAHs (BghiP, InP). The LMW-PAHs (2–3-ring) in the dissolved phase represented on average over 62% of all PAHs, in all the sampling sites. Their abundant concentrations in the water could be justified by their high water solubility and relatively high vapor pressures [40,41,42]. Moreover, the carcinogenic 5–6-ring PAHs were present in low concentrations, accounting for only 17% of the total PAHs. The amount of ΣPAHs in the DP samples (64.3–1429.1 ng L^−1^) was much higher than those found in other rivers of the world (Table 2) as in the Xijiang River [43], in the Yellow River [44], in the Songhua River, China [45], in the Wyre River, England [46], in the Elbe and Weser Rivers, Germany [47], and in the Tiber River, Italy [34,48].However, the PAHs were present in smaller quantities than those found in the Daliao River [49], in the Yellow River [50], in the Songhua River [51], in the Daliao River estuary, China [52], in the Gomti River, India [53], in the Cauca River, Colombia [8], in the Almendares River, Cuba [54], and in the Buffalo River estuary, South Africa [55]. The PAHs concentrations found inthis study are similar to those seen in the Henan Reach of Yellow River, China [56], in the Danube River, Hungary [57], and in the Sarno River [26]. The levels of the reported PAHs compound are relatively high, comparable to those reported for the unpolluted open sea by Berrojalbiz et al. (2011) and Marrucci et al. (2013) (0.7 and 0.4 ng/L, respectively) [58,59].

### 3.2. PAHs Concentrations in Suspended Particulate Matter

Table 1 shows the PAHs concentration ranges in SPM samples: From 149.3 ng L^−1^ in site 8 to 444.9 ng L^−1^ in site 1 (mean value of 264.7 ± 83.3 ng L^−1^). The PAHs amount obtained ranged from 4.05 to 38.9 ng L^−1^ with a mean value of 15.1 ± 8.1 ng L^−1^ for 2-ring PAHs (Nap), from 51.8 to 154.1 ng L^−1^ for 3-ring PAHs (Acy, Ace, Flu, Phe, An), from 39.6 to 181.0 ng L^−1^ for 4-ring PAHs (Fl, Pyr, BaA, Chr), from 26.5 to 103.1 ng L^−1^ for 5-ring PAHs (BbF, BkF, BaP, DahA), and from 17.8 to 66.6 ng L^−1^ for 6-ring PAHs (BghiP, InP). The 4-, 5-, 6-ring PAHs in SPM were present in high concentrations at most sampling sites, with 25%, 20%, and 12% of ΣPAHs, respectively.

HMW-PAHs in SPM samples rised to 57%, while in DP samples was just 38%. In accordance with the PAHs distribution theory, data showed that HMW compounds were mainly sorbed by SPM due to the elevated lipophilicity and poor biodegradation [48,51,57]. According to the partition coefficients (K_p_ = C_SPM_/C_DP_, where C_SPM_ and C_DP_ are the concentrations of analyte in SPM and DP, respectively), HMW-PAHs had a higher affinity to bind with SPM than DP (average value of 0.80, 0.96, and 1.00, respectively for 4-, 5-, 6-ring PAHs).

Table 2 shows the concentration ranges of PAHs found in several rivers around the world. The comparison showed that the PAHs concentrations in SPM samples were higher than those found in the Xijiang River, Yellow River, and Songhua River, China [43,44,45,51], in the Henan Reach of Yellow River, China [56], contrary to those observed in the Daliao River estuary, China [49,52] and in the Sarno River [26], which were higher than those found in this study.

### 3.3. PAHs Concentrations in Sediments

The amount of total PAHs in the sediment samples are shown in Table 1. The data range from 434.8 (site 8) to 872.1 (site 1) ng g^−1^ with a mean value of 659.1 ± 136.9 ng g^−1^. The amount identified ranged from 5.29 to 73.7 ng g^−1^ with a mean value of 24.1 ± 27.5 ng g^−1^ for 2-ring PAHs (Nap), from 42.9 to 186.3 ng g^−1^ for 3-ring PAHs (Acy, Ace, Flu, Phe, An), from 61.7 to 199.7 ng g^−1^ for 4-ring PAHs (Fl, Pyr, BaA, Chr), from 262.7 to 507.1 ng g^−1^ for 5-ring PAHs (BbF, BkF, BaP, DahA), and from 17.5 to 133.2 ng g^−1^ for 6-ring PAHs (BghiP, InP). The 4- and 5-ring PAHs in the sediments were present in high concentrations at most sampling sites, with 37% and 40% of ΣPAHs, respectively. LMW-PAHs concentrations were progressively decreased by dilution due to their high water solubility and easier degradation.

Therefore, HMW compounds had a higher resistance to degradation and could easily reach the sediment due tothe low water solubility, low vapour pressure, and refractory behavior [41,67,68,69].

**Figure 2:**
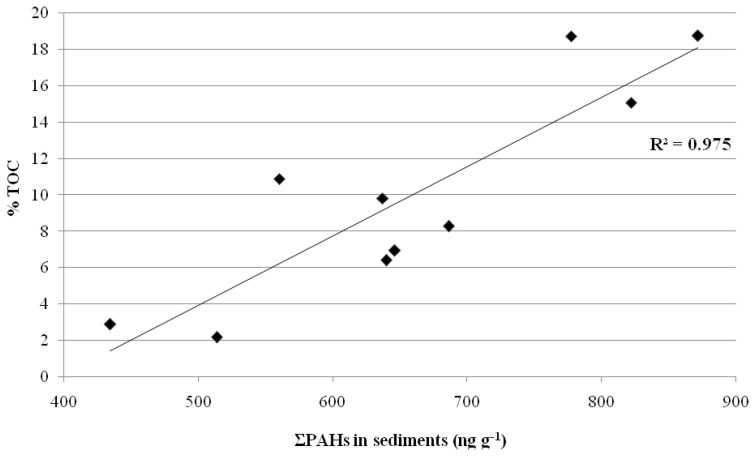
Relationship between the total organic carbon (%TOC) and ΣPAHs in the sediment samples of the Volturno River.

Table 2 shows the concentration ranges of PAHs found in several rivers around the world. The comparison showed that the amount of ΣPAHs in the sediment samples (434.8–872.1 ng g^−1^) was similar to those found in the Yellow River, China [50], in the Erjien River, Taiwan [60], and was greater than the concentration found in the East China Sea, China [61], in the Yellow River and in the Henan Reach of Yellow River, China [44,56], in the Yellow River Estuary, China [62], in the Danube River, Hungary [57],and in Italy, in the Tiber River [34,48,63] and in the Sarno River [26]. However, the amount of ΣPAHs in the sediment samples was lower than the concentration found in the Daliao River, China [49], in the Cocó and Ceará Rivers, Brazil [64], in the Cauca River, Colombia [8], in the Buffalo River Estuary, South Africa [55], in the Ammer River, Germany [9], and in Durance River and Huveaune River, France [65,66]. Low PAHs concentrations can be justified by the low total organic carbon (TOC) (1.1–9.5 mg g^−1^, mean value of 5.1 mg g^−1^) and high content of sand; the association between %TOC and the ΣPAHs is shown in Figure 2. The data showed that there is a positive linear regression between PAHs and TOC in the sediment samples (r = 0.97, *p* < 0.01), as reported by many other studies [8,49,56].

### 3.4. PAHs Seasonal and Spatial Distribution in DP, SPM, and Sediment Samples

The amount of total PAHs found in water and sediment samples at different sampling locations are reported in Table 1. The ratio of the concentration of ΣPAHs in DP samples to that in SPM was higher than one in all sites (average 2.5; SD ± 1.5), as the result of the greater concentrations of PAHs in DP samples rather than in SPM samples for each site and season. These results were also validated by the analysis of the ratio of the individual PAHs and it was possible to observe the same trend obtained from the reports of the sums.

Rather, the ratio of the concentration of ΣPAHs in the sediment samples (ng g^−1^) to that in the SPM samples (ng g^−1^) was below 1 in all sampling locations (average 0.014; range 0.006–0.022; SD ± 0.006), implying that the concentrations of PAHs in SPM samples were higher than those of the sediment samples for each sampling site. Moreover, the results showed that the PAHs concentrations in DPdecreased from July to February, in parallel with the increase in rainfall, which could cause dilution ratio variations. Therefore, the decrease of PAHs concentrations moving from the Volturno River mouthto the Mediterranean Sea is also affected by the high flow in the rainfall season, which results in an even higher dilution ratio.

The lowest concentrations were recorded in the dry season (July), due to the decrease in flow and a greater stagnation of SPM, which led to the shift of PAHs with a greater polarity from SPM to DP.

Finally, the variation in the flow according to the different seasons involved a change in the load and distribution of the PAHs among the DP, SPM, and sediment samples and the higher concentrations of PAHs in SPMs compared to the sediment suggested that the pollution of PAHs which is drained into the Mediterranean Sea is probably due to the fresh input.

The total load of PAHs into the Tyrrhenian Sea was evaluated to estimate the input of PAHs drained from the rainwater outflow, tributary inflow, wastewater treatment plant and industrial effluent discharge, agricultural runoff, atmospheric deposition, and dredged material disposal. The total PAHs loads contribution to the Tyrrhenian Sea from the Volturno River mouth is calculated in about 3158.2 kg/year (calculated by multiplying the average annual flow of 82.1 m^3^/s and the total PAHs average concentrations found at the Volturno River mouth in April, July, November, and February). The total PAHs loads contribution to the Tyrrhenian Sea from the Volturno River mouth is calculated in about 781.6 kg in the spring season (average flow in the spring season, 76.1 m^3^/s, multiplied by the total PAHs concentration found at the Volturno River mouth in April), in about 537.5 kg in the summer season (average flow in the summer season, 40.1 m^3^/s, multiplied by the total PAHs concentration found at the Volturno River mouth in July), in about 1216.5 kg in the autumn season (average flow in the autumn season, 138.1 m^3^/s, multiplied by the total PAHs concentration found at the Volturno River mouth in November), and in about 625.1 kg in the winter season (average flow in the winter season, 104.2 m^3^/s, multiplied by the total PAHs concentration found at the Volturno River mouth in February).

**Figure 3:**
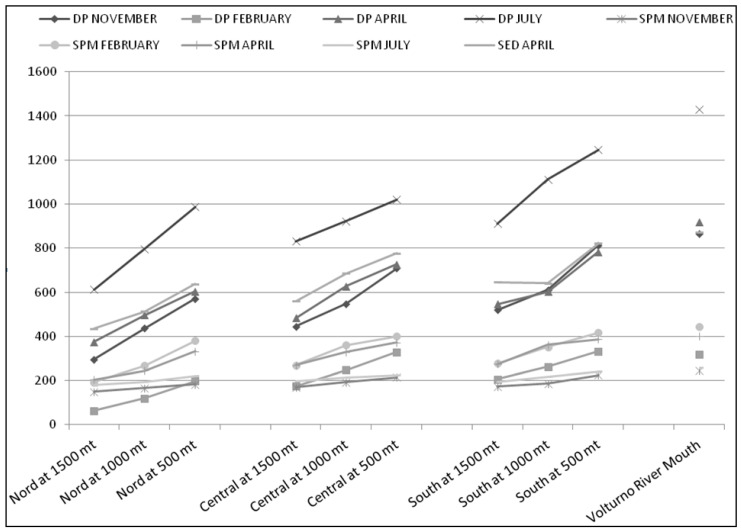
Spatial and temporal concentration of PAHs in the water dissolved phase (DP, ng L^−1^), suspended particulate matter (SPM, ng L^−1^), and sediments (ng g^−1^ dry wt) of the Volturno River and Estuary, Southern Italy.

The spatial distribution of PAHs in DP, SPM, and sediment samples was evaluated and the concentrations of ΣPAHs are shown in Figure 3, with respect tothe different sampling locations and different seasons, with and without rain. Indeed, the level of contamination of PAHs in the water clearly decreased from location 1 to 4. The total PAHs concentrations decreased to 1219.8 ng L^−1^ (DP+SPM mean values of four seasons) at location 1 (Volturno River mouth) to 993.8 ng L^−1^ (DP+SPM mean values of four seasons) at location 2 (500mt from the mouth) to 823.0 ng L^−1^(DP+SPM mean values of four seasons) at location 3 (1000mt from the mouth), and to 668.0 ng L^−1^ (DP+SPM mean values of four seasons) at location 4 (1500mt from the mouth). In the Tyrrhenian Sea, PAHs concentrations were higher near river outflows and much lower in offshore areas (Figure 3).

Figure 3 shows that the PAH concentrations estimated at 500mt of the river outflows were comparable to those at the mouth of the Volturno River, while these were lower at 1000 and 1500mt of the river outflows. In particular, at the mouth of the Volturno, the load of PAHs moves into the Tyrrhenian Sea southwards (Figure 3).

In regards to the data obtained, the trend concentrations showed a decreasing movement from the mouth towards 1500mt at sea. This can depend both on the flow of the river which varies according to the season and on the diluting effect of the sea.

### 3.5. Source Identification

To analyze the origin of PAHs and identify separately petrogenic from pyrolytic inputs, chemical profiling and different diagnostic ratios on isomeric relations were used: An/(An + Phe), Fl/(Fl + Pyr), BaA/(BaA + Chr), and InP/(InP + BghiP) [13,70]. The pyrolytic sources include combustion of fossil fuels, gasoline or diesel fuel vehicles, carbon black, coal tar pitch, asphalt and petroleum cracking, while the petrogenic sources are about crude oil and petrochemicals (gasoline, diesel fuel, kerosene, and lubricating oil). Finally, apart from the pyrolytic or petrogenic source, PAHs can be formed during diagenetic processes, i.e., the formation of sediments from organic material [6]. Each source (i.e., pyrolytic, petrogenic, and diagenetic) provides typical PAH patterns. Typically, HMW compounds with four or more condensed aromatic rings are more abundant in combustion products, while bi- and tricyclic aromatic compounds (LMW) are more abundant in fossil fuels, which are, moreover, dominated by alkylated derivatives [9,10,11].

**Figure 4:**
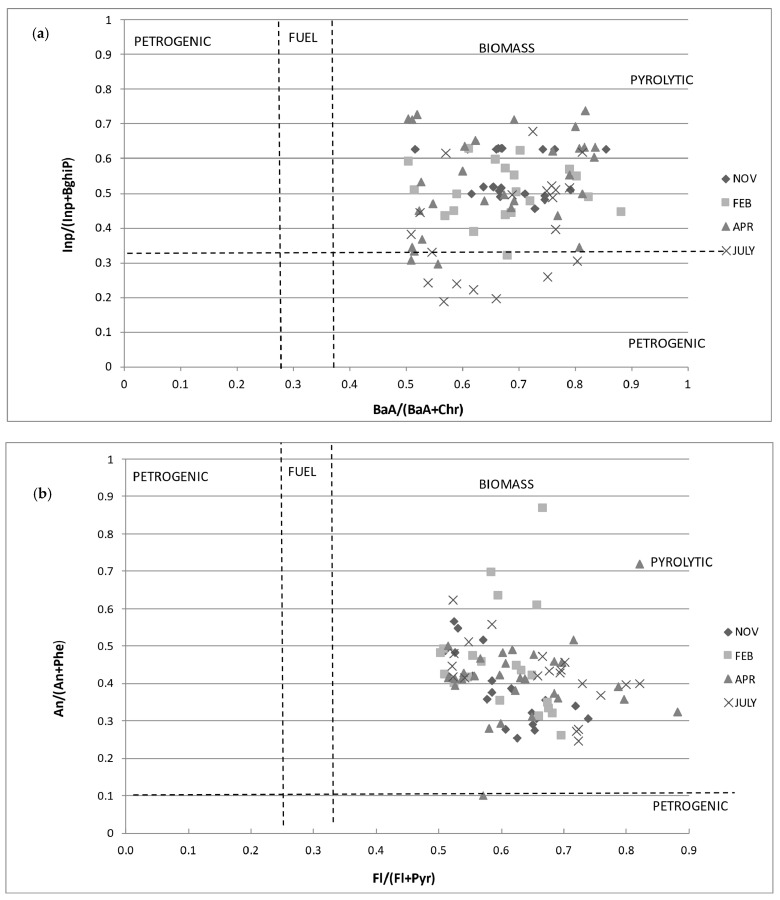
(**a**) Cross plots of the values of Fl/(Fl + Pyr) versus An/(An + Phe) and (**b**) BaA/(BaA + Chr) versus InP/(InP + BghiP) for all the samples data of the Volturno River and its Estuary.

The evaluation of the above ratio indicated the prevalence of PAHs pyrolytic inputs from the Volturno River and its Estuary. In particular, the An/(An + Phe) ratio was >0.1 for all the samples (mean 0.42, 0.40, and 0.47, respectively) and this is ascribed to the origin of PAHs to pyrogenic sources. Moreover, Fl/(Fl+Pyr) ratios can distinguish the petroleum input from combustion processes and discriminate among such sources [13,71]. In particular, Fl/(Fl+Pyr) ratios <0.40 suggest petroleum, ratios from 0.40 to 0.50 are indicative of liquid fossil fuel combustion, and ratios >0.50 are typical for grass, wood, or coal combustion. The ratio Fl/(Fl + Pyr) was >0.5 relatively to all the samples, suggesting the variability of the impacts related to urban traffic emissions and biomass combustion (Figure 4a). BaA/(BaA + Chr) and InP/(InP + BghiP) ratios were >0.35 for water and sediments and these are associated, respectively with vehicular emissions and combustion sources (Figure 4b). In addition, the LMW/HMW ratio was evaluated and it was below 1 for most locations, implying a pyrolytic origin of PAHs (mean 0.85; range 0.09–2.99).

These results reflect the contamination conditions in the Volturno River flatland, which is a dense industrial area. Moreover, a widely documented illegal disposal of urban, toxic, and industrial wastes in the Campania region has occurred [17,72]. The industrial wastes enriched with combustion-derived PAHs are directly discharged into the Volturno River. Although none of the industries present in the Volturno River area exceeded the legal limits in terms of emissions to the atmosphere or industrial discharges as reported by the Piano Regione Campania, the industrial emissions could lead to significant air pollution and the PAHs related to the particulate matter could accumulate into the river over time. In addition to these inputs, some other sources such as the roads on both sides of the river and along the coast, the runoff containing street dust, and municipal wastewater, result in the pattern of pyrolytic origins of PAHs contamination in the area. About that, no other rivers in the area adjacent to that of the Volturno River has been considered with regard to the evaluation of the PAHs and for this reason valid comparisons cannot currently be made. However, some rivers have been taken into consideration for the evaluation of the PAHs, even if they are at greater distances from the Volturno River [33,48,73].

In addition to pyrolytic and petrogenic sources, Per may result from diagenesis processes: A high concentration in the sediment may indicate a natural origin of these compounds as Per is a major diagenetic precursor [6,12,70]. In particular, a Per amount above 10% of the total penta-aromatic isomers suggests a possible diagenetic input, while concentrations below 10% indicate a possible pyrolytic origin of the analyte [12,74]. In the present paper, the concentrations of Per identified in all the sediment samples (range 4.3–15.5 ng g^−1^) contributed less than 2% to the penta-aromatic isomers, suggesting a pyrolytic origin of these compounds.

### 3.6. A Composite Indicator for Water Pollution

**Figure 5:**
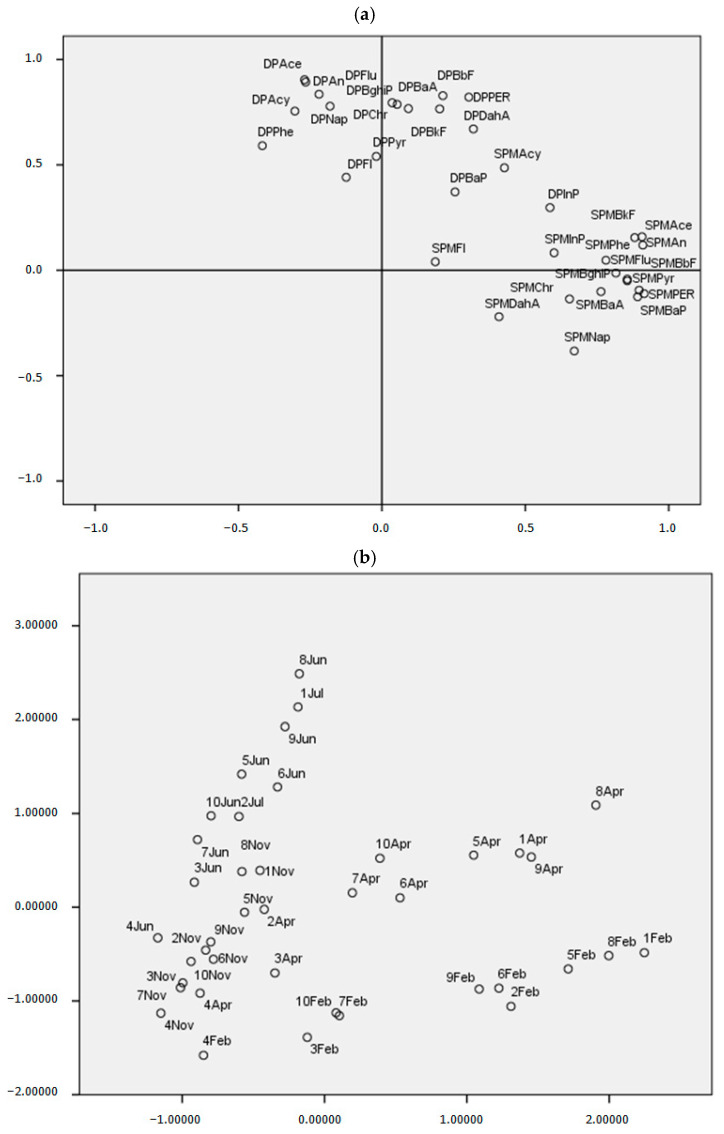
(**a**) Principal component analysis of the DP and SPM data of the Volturno River. Score plot for the first and second principal component. (**b**) Principal component analysis of the DP and SPM data of the Volturno River. The loading plot for the first and second principal component.

In order to formalize a water pollution composite indicator (WP-CI), we analyzed at the same time the dissolved phase (DP) and suspended particulate phase (SPM) samples collected from 10 sites (“Sou1”, “500N2”, “1000N3”, “1500N4”, “500C5”, “1000C6”, “1500C7”, “500S8”, “1000S9”, “1500S10”) during the months of April, July, November, and February. The correlation matrix points out sets of correlated variables and only the first seven highest Eigenvalue are larger than one. However, according to Cattell’s scree test, we considered the first two components that explain the 60.0% (32.6% and 27.4%, respectively) of the total variance. The PCA for this dataset pointed out a clear distinction of the pollution of the two phases and allowed us to define two specific composite indicators (SCIs). In fact, the first factor is characterized by the presence of PAHs belonging to SPM and we named it “SPM—Composite Indicator”; the second factor is defined by the PAHs of the DP, the second factor is called “DP—Composite Indicator”. Looking at the plot of the first two principal components, and making a correlation between the sites and seasons, we observed that the pollution from SPM is higher in February, in the sites 500N2, 1000C6, 1500C7, 1000S9. However, theDP pollution is higher in July at sites 1500C7 e 1500S10 (Figure 5a,b). For each SCI, it is possible to rank the 40 statistical units and finally it is possible to observe the final ranking based on the WP-CI (Table 3). The site that has a lower rate of global pollution in all seasons of the year is the 4, followed by 3, and then 7. However, just in reference to site 7, there is an irregular behavior of the two parties. In fact, while in November, both SPM and DP appear to have a low level of pollution, in other seasons, the two components have a contrasting behavior. The most polluted months are February and April, especially for the SPM component, on the contrary, the least polluted months are July and November, in particular for the SPM component. The month of February, instead, has a tendency of a lower pollution for the DP, on the contrary, July and April are the months most polluted. Based on these results, it can be confirmed that the load and relocation of PAHs between different phases in each sampling site were related to a variation in the flow during the rainy and dry seasons.

### 3.7. Risk Assessment

To estimate the potential adverse effects due to PAHs, the sediment quality guidelines (SQGs) values proposed by Long et al. [75] and by MacDonald et al. [76] were used. Sediment quality guidelines (SQGs) are useful to estimate the contamination of the sediment in the marine ecosystem. Two sets of SQGs, including the ERL/ERM and the TEL/PEL values, were applied in this study to assess the toxic effects of individual PAHs in sediments. These sets are defined as: (i) Effect range low (ERL)/effect range median (ERM) and (ii) the threshold effect level (TEL)/probable effect level (PEL). ERLs and TELs represent chemical concentrations below which the probability of toxicity and other effects are rare, while the ERMs and PELs indicate mid-range above which adverse effects are more likely to occur. ERLs-ERMs and TELs-PELs represent a possible-effects range, within which negative effects would occasionally occur [77]. In the analyzed samples, although some concentrations of PAHs detected are above the TEL and ERL values, the amount of PAHs in the sediment samples was significantly lower than the PEL and ERMvalues (Table 4). In particular, TEL values were exceeded for Acy, Ace, and DahA for all the samples, for Nap and Flu in 30%, and for BaP 80%, suggesting that adverse effects might occasionally occur. The concentrations of individual PAHs do not exceed their respective ERM values, but the ERL values exceeded for Flu in 30%, Ace in 70%, and DahA for all the samples. The results indicated that in certain sites, PAHs may have been found and the environmental integrity was at risk of PAHs in the sediments from the study area.

Although compliance with the European Commission—Environmental Quality Standards (EC-EQS) in surface waters is checked using an annual average of monthly whole water (DP + SPM) concentrations [78], our data showed that the mean values of BaP and BkF + BbF concentration (63.9 and 41.2 ng L^−1^, respectively) were higher than the EQS values (50 and 30 ng L^−1^, respectively), and the mean value of BghiP + InP values (67.4 ng L^−1^) was significantly higher than the EQS value of 2 ng L^−1^, showing that the environmental integrity of the ecosystem was at risk. Moreover, the risk quotient (RQ), the ratio between the measured environmental concentration (MEC) and the predicted no effect concentrations (PNECs), has been calculated. The OSPAR Commission proposed a list of PNECs for several substances, including PAHs. In particular, in the OSPAR Agreement 2014-05, in Table 2, Section 3, PNECs values were reported for single PAHs. According to these values, the ratio between single MEC and PNEC for single PAHs was calculated and as a result, an RQ >1 for most compounds, both for water (sum of DP + SPM) and the sediment, was obtained, confirming that the environmental integrity of the ecosystem was at risk.

## 4. Conclusions

This research is part of a larger project which brings forth fundamental data on the frequency, distribution, and likely sources of PAHs from the Volturno River and its input into the Tyrrhenian Sea (Central Mediterranean Sea), Southern Italy. LMW-PAHs were abundantly present in the water samples, while in sediment samples, the predominant class were high molecular weight PAHs. The concentration levels of PAHs in DP, SPM, and sediment phases were remarkably different amongst the sampling sites. Contaminant discharges of PAHs into the sea showed that this river should account as one of the main contribution sources of PAHs to the Central Mediterranean Sea. A water pollution composite indicator (WP-CI) as well asan individual diagnostic PAHs ratio revealed that the main PAHs source was pyrolytic and suggested that the majority of this pollution derived for the most part from vehicle traffic and combustion processes. Regarding the risk assessment, even if the concentration of many single PAHs in a number of stations were above ERL and/or TEL (and below ERM and/or PEL), which would on occasion yield negative environmental consequences, the European Commission—Environmental Quality Standards (EC-EQS) and the risk quotient (RQ) indicated that the integrity of this area is possibly at risk. Therefore, the Volturno River waters should be continuously kept under monitor observation, as the PAHs could lead to negative consequences on its aquatic ecosystems and organisms.

## Figures and Tables

**Table 1:** Description of the sampling sites and concentration of polycyclic aromatic hydrocarbons (PAHs) in the water dissolved phase (DP), suspended particulate matter (SPM), and the sediments of the Volturno River, Southern Italy.

Sampling Location			ΣPAHs								
Site Number Identification	Site Characteristics	Site Location	Dissolved Phase (ng L^−1^)				Particulate Phase (ng L^−1^) (ng g^−1^ dry wt)				Sediments (ng g^−1^ dry wt)
			Apr	Jul	Nov	Feb	Apr	Jul	Nov	Feb	Apr
1 (river water)	Volturno River Source	40°48′54.03″ N 14°36′45.36″ E	919.8	1429.1	865.1	318.1	401.1 (49,334.1)	257.1 (26,324.6)	243.8 (21,499.3)	444.9 (155,032)	872.1
2 (sea water)	River Mouth at 500mt North	40°46′42.73″ N 14°34′00.48″ E	604.0	987.5	571.1	197.8	332.3 (35,892.9)	221.8 (118,021.2)	183.3 (5907.4)	381.0 (3253.1)	637.2
3 (sea water)	River Mouth at 500mt Central	40°46′00.34″ N 14°33′10.68″ E	726.9	1020.9	708.8	329.4	372.3 (94,015.7)	225.1 (79,558.8)	214.5 (68,758.0)	400.9 (134,546)	777.5
4 (sea water)	River Mouthat 500mt South	40°43′42.62″ N 14°28′07.89″ E	783.3	1246.2	813.3	332.4	387.9 (38,000.5)	241.8 (226,017.2)	225.0 (6516.6)	417.2 (3902.2)	822.2
5 (sea water)	River Mouth at 1000mt North	40°43′40.11″ N 14°28′06.45″ E	496.6	797.4	436.2	120.8	243.9 (32,792.5)	194.3 (13,512.9)	167.2 (12,661.5)	267.7 (2644.7)	514.1
6 (sea water)	River Mouth at 1000mt Central	40°43′42.46″ N 14°28′05.03″ E	627.3	923.6	548.9	248.1	328.7 (114,934.8)	214.7 (71,336)	193.2 (67,336.9)	361.1 (118,028)	686.5
7 (sea water)	River Mouth at 1000mt South	40°43′45.09″ N 14°28′05.17″ E	602.7	1111.2	611.5	262.1	361.7 (41,484.5)	218.7 (14,888.4)	185.7 (9221.1)	351.7 (2946.8)	640.1
8 (sea water)	River Mouth at 1500mt North	40°43′35.68″ N 14°28′02.94″ E	375.4	612.4	295.2	64.3	204.2 (20,348.5)	179.0 (10,706.3)	149.3 (7564.9)	192.3 (60,683.0)	434.8
9 (sea water)	River Mouth at 1500mt Central	40°43′42.25″ N 14°27′59.97″ E	482.8	831.7	445.3	174.9	268.6 (86,952.9)	197.9 (66,193.6)	169.5 (60,767.3)	269.1 (91,871)	560.4
10 (sea water)	River Mouth at 1500mt South	40°43′49.26″ N 14°27′59.82″ E	545.8	911.9	519.5	206.0	275.0 (97,525.2)	194.1 (16,159.0)	173.1 (4485.7)	277.9 (97,859.7)	646.3

**Table 2:** Concentration ranges and mean value of PAHs in the water dissolved phase (DP), suspended particulate matter (SPM), and sediments from recent studies of different rivers and estuaries in the world.

Area	References	Number PAHs	Range ∑PAHs	Mean ∑PAHs
Water (ng L^−1^)				
Xijiang River, China	Deng et al. [43]	15	21.7–138.0	-
Yellow River, China	Li et al. [44]	15	179.0–369.0	248.2
Daliao River, China	Guo et al. [49]	18	570.2–2318.6	-
Henan Reach of Yellow River, China	Sun et al. [56]	16	144.3–2361.0	662.0
Songhua River, China	Ma et al. [45]	15	14.0–161.0	33.9
Yellow River, China	Zhao et al. [50]	16	548.0–2598.0	1375.0
Songhua River, China	Zhao et al. [51]	16	163.5–2746.2	934.6
Daliao River estuary, China	Zheng et al. [52]	16	71.1–4255.4	748.8
Gomti River, India	Malik et al. [53]	16	60–84,210.0	10,330.0
Cauca River, Colombia	Sarria-Villa et al. [8]	12	52.1–12,888.2	2344.5
Almendares River, Cuba	Santana et al. [54]	14	836.0–15,811.0	2512.0
Buffalo River Estuary, South Africa	Adeniji et al. [55]	16	ND-24,910	-
Wyre River, England	Moeckel et al. [46]	28	2.7–20.0	-
Elbe and Weser Rivers, Germany	Siemers et al. [47]	16	10.0–40.0	-
Danube River, Hungary	Nagy et al. [57]	16	25.0–1208.0	122.6
Tiber River, Italy	Patrolecco et al. [48]	6	23.9–72.0	43.4
Tiber River, Italy	Montuori et al. [34]	17	1.75–607.48	90.46
Sarno River, Italy	Montuori and Triassi [26]	17	12.4–2321.1	739
Xijiang River, China	Deng et al. [43]	15	1.4–58.1	29.8
Yellow River, China	Li et al. [44]	13	54.0–155.0*	-
Daliao River, China	Guo et al. [49]	18	151.0–28,483.8	-
Henan Reach of Yellow River, China	Sun et al. [56]	16	506.6–10,510.0*	4100.0 *
Songhua River, China	Ma et al. [45]	15	9.21–83.1	26.4
Yellow River, China	Zhao et al. [50]	16	1502.0–11,562.0*	5591.0 *
Daliao River estuary, China	Zheng et al. [52]	16	1969.9–11,612.2	4015.7
Tiber River, Italy	Patrolecco et al. [48]	6	37.6–353.0	
Tiber River, Italy	Montuori et al. [34]	17	4.53–473.39	111.51
Sarno River, Italy	Montuori and Triassi [26]	17	6.1–778.9	-
Yellow River, China	Li et al. [44]	13	31.0–133.0	76.8
Daliao River, China	Guo et al. [49]	18	102.9–3419.2	-
Henan Reach of Yellow River, China	Sun et al. [56]	16	16.4–1358.0	182.0
Yellow River, China	Zhao et al. [50]	16	181.0–1583.0	810.0
Erjien River, Taiwan	Wang et al. [60]	16	22.0–28,622.0	737.0
East China Sea, China	Zhao et al. [61]	16	57.5-364.5	166.2
Yellow River Estuary, China	Liu et al. [62]	15	89.5–208.0	140.5
Tiber River, Italy	Minissi et al. [63]	13	4.5–652.2	-
Cocó and Ceará Rivers, Brazil	Cavalcante et al. [64]	17	3.0–2234.8	-
Cauca River, Colombia	Sarria-Villa et al. [8]	12	ND-3739.0	1028.0
Buffalo River Estuary, South Africa	Adeniji et al. [55]	16	ND-7792	-
Ammer River, Germany	Liu et al. [9]	16	112.0–22,900.0	8770.0
Danube River, Hungary	Nagy et al. [57]	16	8.3–1202.5	170.0
Huveaune River, France	Kanzari et al. [65]	16	571.7–4234.9	1966.00
Durance River, France	Kanzari et al. [66]	16	57.0–1528.0	-
Ría de Arousa, Spain	Peréz-Fernández et al. [6]	35	45.0–7901.0	-
Tiber River, Italy	Patrolecco et al. [48]	6	157.8–271.6	215.2
Tiber River, Italy	Montuori et al. [34]	17	36.21–545.60	155.26
Sarno River, Italy	Montuori and Triassi [26]	17	5.5–678.6	266.9
This study	DP	17	64.3–1429.1	602.6 ± 319.3
		
	SPM		143.3–444.9	264.7 ± 83.3
		
	Sediment		434.8–872.1	659.1 ± 136.9

* ng/g; ND: Not detectable.

**Table 3:** Rankings based on SCIs and WP-CI according to these thresholds, (1): Normalized score > 0:60, (2): Normalized score > 0:30 and < 0:60, (3): Normalized score < 0:30.

	− High Pollution +													
**SPM Specific Composite Indicator**	07-apr	10-apr	06-apr	05-apr	09-feb	06-feb	02-feb	01-apr	09-apr		05-feb	08-apr	08-feb	01-feb
**DP Specific Composite Indicator**	10-apr	09-apr	05-apr	01-apr	07-jul	02-jul	10-jul	08-apr	06-jul		05-jul	09-jul	01-jul	08-jul
**WP Composite Indicator**	07-apr	09-jul	06-apr	01-jul	10-apr	05-feb	08-jul	05-apr	08-feb		01-feb	01-apr	09-apr	08-apr

	**− Medium Pollution +**													
**SPM Specific Composite Indicator**	02-jul	05-jul	08-nov	05-nov	01-nov	02-apr	03-apr	06-jul	09-jul	01-jul	08-jul	03-feb	10-feb	07-feb
**DP Specific Composite Indicator**	10-nov	06-nov	08-feb	01-feb	02-nov	09-nov	04-jul	05-nov	02-apr	06-apr	07-apr	03-jul	08-nov	01-nov
**WP Composite Indicator**	03-feb	05-nov	10-feb	07-feb	10-jul	08-nov	02-apr	02-jul	01-nov	05-jul	09-feb	02-feb	06-feb	06-jul

	**− Low Pollution +**													
**SPM Specific Composite Indicator**	04-jul	04-nov	03-nov	07-nov	10-nov	03-jul	07-jul	04-apr	04-feb		02-nov	09-nov	10-jul	06-nov
**DP Specific Composite Indicator**	04-feb	03-feb	07-feb	04-nov	10-feb	02-feb	04-apr	09-feb	06-feb		03-nov	07-nov	03-apr	05-feb
**WP Composite Indicator**	04-nov	03-nov	07-nov	04-feb	04-jul	04-apr	10-nov	03-jul	02-nov		09-nov	06-nov	07-jul	03-apr

**Table 4:** A comparison of the threshold effect level (TEL), probable effect level (PEL), effect range low (ERL), and effect range median (ERM) guideline values (µg Kg^−1^) for polycyclic aromatic hydrocarbons and data found in the Volturno River, Southern Italy.

	PAHs																
	Nap	Acy	Ace	Flu	Phe	An	Fl	Pyr	BaA	Chr	BbF	BkF	BaP	DahA	BghiP	InP	∑PAHs
TEL ^a^	34.6	5.87	6.71	21.2	86.7	46.9	113	153	74.8	108	-	-	88.8	6.22	-	-	1684
Samples percentage over the TEL	30	100	100	30	0	0	0	0	0	0			80	100			0
PEL ^a^	391	128	88.9	144	544	245	1494	1398	693	846	-	-	763	135	-	-	16,770
Samples percentage over the PEL	0	0	0	0	0	0	0	0	0	0			0	10			0
ERL ^a^	160	44	16	19	240	85	600	665	261	384	-	-	430	63.4	-	-	4022
Samples percentage over the ERL	0	0	0	0	0	0	0	0	0	0			0	100			0
ERM ^a^	2100	640	500	540	1500	1100	5100	2600	1600	2800	-	-	1600	260	-	-	44,792
Samples percentage over the ERM	0	0	0	0	0	0	0	0	0	0			0	0			0

^a^ MacDonald et al. [76]. ^b^ Long et al. [75].

## Data Availability

The datasets obtained and analyzed in the current study are available from the corresponding author on a reasonable request.

## Supplementary Materials

The following are available online at https://www.mdpi.com/1660-4601/18/4/1383/s1, Table S1: Environmental data of water samples from sampling sites.

## Abbrevations

PAHs: Polycyclic aromatic hydrocarbons; HMW: High molecular weight; LMW: Low molecular weight; SPM: Suspended particulate matter; DP: Dissolved phase; SIM: Single ion monitoring mode; USEPA: US Environmental Protection Agency; Nap: Naphthalene; Acy: Acenaphthylene; Ace: Acenaphthene; Flu: Fluorene; Phe: Phenanthrene; An: Anthracene; Fl: Fluoranthene; Pyr: Pyrene; BaA: Benzo[a]anthracene; Chr: Chrysene; BbF: Benzo[b]fluoranthene; BkF: Benzo[k]fluoranthene; BaP: Benzo[a]pyrene; DahA: Dibenzo[a,h]anthracene; BghiP: Benzo[ghi]perylene; InP: Indeno[1,2,3-cd]pyrene; Per: Perylene; LOD: Limit of detection; LOQ: Limit of quantification; PCA: Principal component analysis; TOC: Total organic carbon; WP-CI: Water pollution composite indicator; SQGs: Sediment quality guidelines; ERL: Effect range low; ERM: Effect range median; TEL: Threshold effect level; PEL: Probable effect level; EQS: Environmental quality standards; RQ: Risk quotient; MEC: Measured environmental concentration; PNECs: Predicted no effect concentrations; OSPAR: Protection of the Marine Environment of the North-East Atlantic.
