# Supplementary material for: Estimation of Polycyclic Aromatic Hydrocarbons Pollution in Mediterranean Sea from Volturno River, Southern Italy: Distribution, Risk Assessment and Loads
Source: Int J Environ Res Public Health. 2021 Feb 3;18(4):1383. doi: 10.3390/ijerph18041383 (PMC7913333; doi:10.3390/ijerph18041383)
Supplement: Supplementary file 1 [file ijerph-18-01383-s001.pdf]

International Journal of
Environmental Research and Public Health

MDPI

Estimation of Polycyclic Aromatic Hydrocarbons pollution in Mediterranean Sea from Volturno River, Southern Italy: distribution, risk assessment and loads.

Paolo Montuori[1]*, Elvira De Rosa[1], Fabiana Di Duca[1], Donatella Paola Provvisiero[1], Pasquale Sarnacchiaro[2], Antonio Nardone[1], Maria Triassi[1]

[1]Department of Public Health, "Federico II" University, Via Sergio Pansini nº 5, 80131 Naples, Italy
[2]Department of Law and Economics, University of Roma "Unitelma Sapienza", Viale Regina Elena 295, Rome 0016, Italy

Table S1. Environmental data of water samples from sampling sites.

**Site Number Identification**	**Sampling Site**	pH				Conducibility (mS/cm)				Temperature (°C)				Salinity (‰)				Fresh and Sea water ratio			
		Apr	Jul	Nov	Feb	Apr	Jul	Nov	Feb	Apr	Jul	Nov	Feb	Apr	Jul	Nov	Feb	Apr	Jul	Nov	Feb
**1 (river water)**	**Volturno River Source**	7.4	7.9	7.7	7.3	15.2	14.3	10.1	9.8	20.6	24.6	18.5	15.1	9.8	8.4	6.6	6.9	0.26	0.22	0.17	0.18
2 (sea water)	River Mouth at 500mt North	7.0	7.2	7.3	7.2	17.2	18.6	11.5	16.3	22.4	23.9	17.6	16.3	10.7	11.3	7.8	11.7	0.28	0.30	0.21	0.31
3 (sea water)	River Mouth at 500mt Central	7.5	7.5	7.8	6.9	16.6	15.5	10.2	13.5	20.6	23.5	19.2	14.5	10.7	9.3	6.6	10.0	0.28	0.24	0.17	0.26
4 (sea water)	River Mouth at 500mt South	7.1	7.5	7.0	7.5	12.8	15.3	8.0	10.9	19.8	24.3	18.0	14.9	8.3	9.0	5.2	7.8	0.22	0.24	0.14	0.21
5 (sea water)	River Mouth at 1000mt North	6.9	7.4	6.8	7.3	20.4	21.8	19.3	16.2	21.4	22.8	18.3	15.2	13.2	13.7	13.4	11.9	0.35	0.36	0.35	0.31
6 (sea water)	River Mouth at 1000mt Central	7.8	7.0	7.5	7.0	24.9	21.7	22.0	18.5	18.9	24.7	19.0	15.5	17.4	13.1	15.2	13.7	0.46	0.34	0.40	0.36
7 (sea water)	River Mouth at 1000mt South	7.7	7.1	7.3	7.6	23.2	25.2	21.9	20.6	22.3	23.2	17.9	14.6	14.9	16.0	15.5	15.7	0.39	0.42	0.41	0.41
8 (sea water)	River Mouth at 1500mt North	7.0	7.6	7.1	6.8	27.4	35.9	32.8	29.4	21.4	24.9	18.8	15.4	18.2	22.7	23.6	22.8	0.48	0.60	0.62	0.60
9 (sea water)	River Mouth at 1500mt Central	7.2	7.8	7.9	7.3	32.1	36.1	31.4	28.9	22.1	25.1	19.6	14.9	21.4	22.7	22.1	22.6	0.56	0.60	0.58	0.59
10 (sea water)	River Mouth at 1500mt South	7.4	7.7	7.2	7.8	31.6	33.3	31.0	30.4	19.6	24.3	19.5	15.4	22.2	21.2	21.8	23.6	0.58	0.56	0.57	0.62
